# Reply: Ablating atrial fibrillation for tricuspid valve

**DOI:** 10.1016/j.xjon.2022.01.010

**Published:** 2022-01-20

**Authors:** Joon Bum Kim

**Affiliations:** Department of Thoracic and Cardiovascular Surgery, Asan Medical Center, University of Ulsan College of Medicine, Seoul, South Korea

Reply to the Editor:

**Figure:**
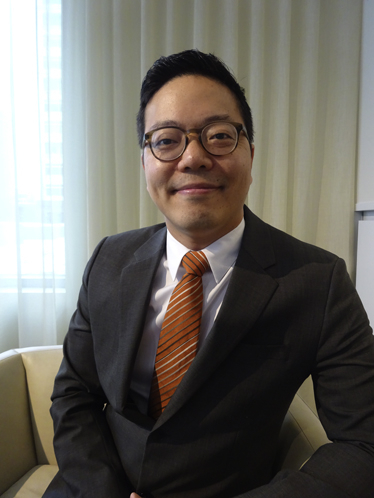

The author reported no conflicts of interest.

The *Journal* policy requires editors and reviewers to disclose conflicts of interest and to decline handling or reviewing manuscripts for which they may have a conflict of interest. The editors and reviewers of this article have no conflicts of interest.

I appreciate Yegorovich and colleagues for their interest in our paper^1^ published in the *Journal* recently, which sought to explore the impact of concomitant tricuspid valve (TV) repair in mild functional tricuspid regurgitation (TR) in rheumatic mitral valve surgery, and we used observational data of 1208 patients retrieved from 2 large-volume centers. In our results, we observed that the addition of TV repair did significantly impact either clinical end points or the progression of TR over a long-term (up to approximately 15 years); however, it is interesting to observe that omission of ablation of atrial fibrillation (AF) 4- to 5-fold increased the risk of developing severe TR. These findings suggested that ablation of AF is more important than repairing TV to prevent worsening of TR in the given settings. Coming back to the comments raised by Yegorovich and colleagues, I summarize their key questions into 3 to follow and answer them one by one.

1. “If you have AF surgical correction carried out in the study group, how were the postoperative outcomes compared with isolated valve disease treatment?”

Among the 1208 subject patients, 557 patients (46.1%) had AF at baseline, and among these, 78.6% (n = 358) underwent concomitant AF ablation whereas the others did not (21.4%). When compared, those who underwent concomitant AF ablation had a significantly lower risk of developing severe TR later on (hazard ratio, 0.16; 95% confidence interval, 0.07-0.35; *P* < .001), although we did not obtain significantly reduced incidences of hard clinical end points (death, heart failure). Through series of our publications on the relevant issue, however, we have constantly shown that concomitant AF ablation during heart valve surgery is likely beneficial in terms of (1) improved survival, (2) reduced thromboembolic risks, (3) improved left ventricular function, and (4) functional durability of TV throughout various risk cohorts, including those with high risk.^2^, ^3^, ^4^, ^5^ These results are now undergoing validation from a nationwide dataset of South Korea covering >17,000 patients, which we believe will be shared in public in the near future.

2. “How common was severe pulmonary hypertension (PHT) in the patients you studied?”

The average level of systolic pulmonary artery pressure was approximately 40 mm Hg without a significant intergroup difference, demonstrating that mild-to-moderate PHT was prevailing, but we estimate that severe PHT (defined by systolic pressure >55 mm) was not so common, 10% to 20%. This is may be because we included only patients with mild TR. We did not also see temporal trends of PHT over time in our particular study depending on TV repair and on AF ablation, and this will be an interesting issue to be studied further.

3. “Do you think the use of radiofrequency denervation is a reasonable method of surgical correction of PHT?”

Honestly, as we have not enough knowledge and experience on this procedure and its impact on outcomes, we are in a position to learn about this from experts like Yegorovich and colleagues, and I read their recent paper focusing on this issue with great interest.^6^ Although the paper has inherent limitations of observational studies bearing selection bias, there seems to be potential in this pulmonary artery denervation to investigate further to see whether this may improve rhythm outcomes and thereby help reverse PHT.
